# Supplementary material for: Hybrid architectures for terahertz molecular polaritonics
Source: Nat Commun. 2024 May 24;15:4427. doi: 10.1038/s41467-024-48764-6 (PMC11126624; doi:10.1038/s41467-024-48764-6)
Supplement: Supplementary file 1 — Supplementary Information [file 41467_2024_48764_MOESM1_ESM.pdf]

Supplementary Information: Hybrid architectures for terahertz molecular polaritonics

A. Jaber,[1,2] M. Reitz,[2,3,4] A. Singh,[1,2] A. Maleki,[1,2] Y. Xin,[5] B. T. Sullivan,[5] K. Dolgaleva,[1,2,6] R. W. Boyd,[1,2,6,7] C. Genes,[2,3,4] and J.-M. Ménard[1,2,6]

[1] *Department of Physics, University of Ottawa, Ottawa, ON K1N 6N5, Canada*
[2] *Max Planck Centre for Extreme and Quantum Photonics, Ottawa, ON K1N 6N5, Canada*
[3] *Max Planck Institute for the Science of Light, D-91058 Erlangen, Germany*
[4] *Department of Physics, Friedrich-Alexander-Universität Erlangen-Nürnberg, D-91058 Erlangen, Germany*
[5] *Iridian Spectral Technologies Ltd., Ottawa, ON K1G 6R8, Canada*
[6] *School of Electrical Engineering and Computer Science, University of Ottawa, Ottawa, ON K1N 6N5, Canada*
[7] *University of Rochester, Rochester, NY 14627, USA*

CONTENTS

S1. Response of a plasmonic MS 1

S2. FDTD simulation of the MS field distribution 2

S3. Transfer matrix theory 3

S4. Coupled-dipoles approach for glucose-MS coupling 4

S5. Constituent transmission curves for H2 6

S6. Empty hybrid cavity 6

S7. Glucose filling fraction 8
A. Overfilling a metasurface 10

S8. Glucose spray coating 10

References 11

S1. RESPONSE OF A PLASMONIC MS

We provide here a quick review of the reflection and transmission properties of two-dimensional MSs as widely covered in the literature, largely following Ref. [S1]. We consider a square array of (ideal, point-like) dipoles with resonance frequency $\omega_0 = ck_0 = 2\pi c/\lambda_0$ (wavenumber k_0, resonance wavelength λ_0), located in the xy plane at positions $\mathbf{r}_j$ with lattice constant a. Each scatterer responds with its induced dipole moment $\mathbf{p}_j = \boldsymbol{\alpha}_\mathrm{p}\mathbf{E}(\mathbf{r}_j)$, determined by its polarizability tensor $\boldsymbol{\alpha}_\mathrm{p}$ and the electric field acting on it and produces a field of $\mathbf{E}^j_\mathrm{dip}(\mathbf{R}) = \mathbf{G}(\mathbf{R}-\mathbf{r}_j)\mathbf{p}_j$ at position $\mathbf{R}$ governed by the free space Green's tensor (evaluated at the dipole resonance) [S1, S2]

$$\mathbf{G}(\mathbf{R}) = (k_0^2 + \nabla\otimes\nabla)\frac{e^{ik_0|\mathbf{R}|}}{|\mathbf{R}|}. \tag{S.1}$$

The self-consistent dipole moment of the jth element is given by

$$\mathbf{p}_j = \boldsymbol{\alpha}_\mathrm{p}\left[\mathbf{E}_\mathrm{in}(\mathbf{r}_j) + \sum_{j'\neq j}\mathbf{G}(\mathbf{r}_j - \mathbf{r}_{j'})\mathbf{p}_{j'}\right], \tag{S.2}$$

which has contributions from the incident field as well as the re-radiated fields from all surrounding dipoles. Let us consider a quasi-infinite array under normal, plane wave illumination $\mathbf{E}_\mathrm{in}(\mathbf{R}) = \mathbf{E}_\mathrm{in}e^{ik_\ell z}$ with wavenumber k_ℓ. In this

case, all dipoles will respond identically, i.e., $\mathbf{p}_j = \mathbf{p}$, yielding a straightforward solution to the above equation as

$$\mathbf{p} = \frac{\mathbf{E}_{\rm in}}{\boldsymbol{\alpha}_{\rm p}^{-1} - \widetilde{\mathbf{G}}(0)}, \tag{S.3}$$

where $\widetilde{\mathbf{G}}(0) = \sum_{j\neq 0} \mathbf{G}(\mathbf{r}_j - \mathbf{r}_0)$ corresponds to the sum of the Green's tensor over all lattice vectors (w.r.t. some arbitrary lattice vector $\mathbf{r}_0$). More generally, for non-normal incidence of the incoming light, this extends to the lattice Fourier transform of the Green's tensor evaluated at the in-plane quasimomentum $\mathbf{q}$: $\widetilde{\mathbf{G}}(\mathbf{q}) = \sum_{j\neq 0} \mathbf{G}(\mathbf{r}_j) e^{-i\mathbf{q}\cdot\mathbf{r}_j}$. The total electric field is obtained as

$$\mathbf{E}(\mathbf{R}) = \mathbf{E}_{\rm in}(\mathbf{R}) + \mathbf{p}\sum_j \mathbf{G}(\mathbf{R} - \mathbf{r}_j), \tag{S.4}$$

requiring the estimate of the sum of all dipole-radiated fields. This sum over the real-space lattice can be turned into a sum over the reciprocal lattice via Poisson's summation formula. For subwavelength arrays $a < \lambda_0$, this expression greatly simplifies as, in the far-field, only a single term survives while all others are evanescent. In addition, only considering a single polarization component (e.g., all dipoles pointing along x), the far field along the z direction is obtained as (assuming $k_0 \approx k_\ell$)

$$E^{\rm far}(z) = E_{\rm in}\left[e^{ik_\ell z} + r_m e^{ik_\ell |z|}\right], \tag{S.5}$$

with the complex reflection coefficient of the MS

$$r_m = \frac{2\pi i k_0/a^2}{1/\alpha_{\rm p}^{xx} - \widetilde{G}_{xx}(0)}, \tag{S.6}$$

while the complex transmission is obtained as $t_m = 1 + r_m$. The frequency-dependent reflectivity can be rewritten in a more intuitive Lorentzian form as

$$r_m(\omega) = -i\frac{\gamma + \widetilde{\gamma}(0)}{(\omega - \omega_0 - \widetilde{\Omega}(0)) + i(\gamma + \widetilde{\gamma}(0))}, \tag{S.7}$$

where we defined the MS-induced frequency shift $\widetilde{\Omega}(0)$ and decay rate modification $\widetilde{\gamma}(0)$ as

$$\widetilde{\gamma}(0) = \frac{3\gamma}{2k_0^3}\mathrm{Im}\left[\widetilde{G}_{xx}(0)\right] = \gamma\frac{3}{4\pi}\left(\frac{\lambda_0}{a}\right)^2 - \gamma, \tag{S.8a}$$

$$\widetilde{\Omega}(0) = -\frac{3\gamma}{2k_0^3}\mathrm{Re}\left[\widetilde{G}_{xx}(0)\right]. \tag{S.8b}$$

The last step in Eq. (S.8a) holds for subwavelength arrays $a < \lambda_0$. Here, we have assumed non-absorbing dipoles with a polarizability of (in cgs units)

$$\alpha_{\rm p}^{xx}(\omega) = -\frac{3}{16\pi^3}\lambda_0^3\frac{\gamma}{(\omega - \omega_0) + i\gamma}, \tag{S.9}$$

where γ is the radiative linewidth of a single dipole. In the following, we denote the MS linewidth as $\gamma_m = \gamma + \widetilde{\gamma}(0)$ and the MS resonance frequency as $\omega_m = \omega_0 + \widetilde{\Omega}(0)$. Note that the above treatment is in principle only valid for particle sizes much smaller than the wavelength of the incident light. For larger particle sizes, electrodynamic corrections have to be taken into account (for details see e.g., Refs. [S3, S4]).

S2. FDTD SIMULATION OF THE MS FIELD DISTRIBUTION

To investigate the evanescent nature of the plasmonic mode volume of the MS, we utilize a FDTD simulation to monitor the electric field distribution. Using the geometry of the cross shown in Fig. 3 of the manuscript with periodic boundary conditions, we can simulate the response of the MS. Figs. S1(a) and (b) show the spatial field distribution of a cross element at the plasmonic resonance frequency in the xy and xz planes, respectively. The incident pulse injected

into the simulated structure is linearly polarized along the x axis. We then investigate the evanescent field strength in the glucose along the z-axis. Fig. S1(c) shows the absolute field calculated at the position ($x = 41.5\,\mu$m, $y = 0\,\mu$m), which is representative of the overall values obtained numerically at different xy positions above the metasurface.

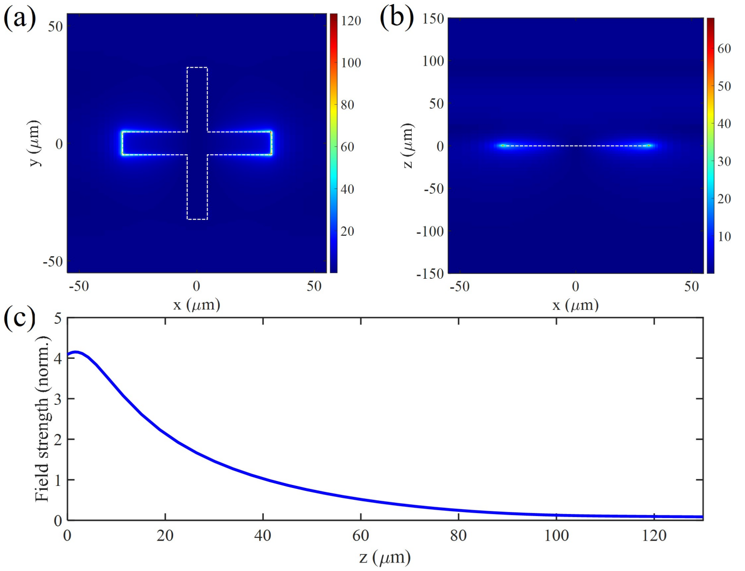

FIG. S1. FDTD simulations of field distribution at the air/substrate/MS interface (shown is a single plasmonic element) (a) in the xy plane ($z = 0\,\mu$m) and (b) in the xz plane ($y = 0\,\mu$m) spanning $150\,\mu$m above and below the interface. (c) Evanescent field strength monitored along z at a position ($x = 41.5\,\mu$m, $y = 0\,\mu$m), which is chosen because it shows a distribution representative of the overall field contained within the xy plane above the metasurface (in glucose).

S3. TRANSFER MATRIX THEORY

Transfer matrix theory is a simple yet powerful method to obtain the transmission and reflection coefficients and thereby the spectral response of layered systems [S5, S6]. The method connects the field amplitudes of left ($-$) and right ($+$) travelling waves on the left of the structure $(E_L^-, E_L^+)^\top$ to the left- and right travelling waves on the right $(E_R^-, E_R^+)^\top$ via the transfer matrix $\mathbf{T}$ such that

$$\begin{pmatrix} E_L^- \\ E_L^+ \end{pmatrix} = \mathbf{T} \begin{pmatrix} E_R^- \\ E_R^+ \end{pmatrix}. \tag{S.10}$$

Assuming a unit input field entering the system from the left $E_L^+ = 1$, and no input field from the right, $E_R^- = 0$, the transmission coefficient is simply obtained as the inverse of the last entry of the transfer matrix $t = 1/T_{22}$. The free propagation through a dispersive medium with index of refraction $n(\omega)$ and length d is described by the matrix

$$\mathbf{T}_f = \begin{pmatrix} e^{i\omega n(\omega)d/c} & 0 \\ 0 & e^{-i\omega n(\omega)d/c} \end{pmatrix}, \tag{S.11}$$

while the action of a thin mirror or MS is described by

$$\mathbf{T}_m = \begin{pmatrix} 1 + i\zeta_m(\omega) & i\zeta_m(\omega) \\ -i\zeta_m(\omega) & 1 - i\zeta_m(\omega) \end{pmatrix}. \tag{S.12}$$

Here, the quantity $\zeta_m(\omega) = -ir_m(\omega)/t_m(\omega)$ is called the polarizability of the mirror which describes the ratio between the complex reflection and transmission coefficients. This is not to be confused with the polarizability from section S1. While this is a strongly frequency-dependent quantity for the MS, we assume the polarizability of the gold mirrors to be constant within the considered frequency range. Assuming a Lorentzian reflectivity for the MS (compare with Eq. (S.7)), given by $r_m(\omega) = -i\gamma_m/[(\omega - \omega_m) + i(\gamma_m + \gamma_l)]$ and including an additional nonradiative loss channel γ_l,

the polarizability of the MS simply expresses as

$$\zeta_m(\omega) = \frac{\gamma_m}{(\omega_m - \omega) - i\gamma_l}. \tag{S.13}$$

In addition, the Fresnel losses at the interface between two layers j and $j+1$ can be taken into account by the transfer matrix

$$\mathbf{T}_{j,j+1} = \frac{1}{t_{j,j+1}} \begin{pmatrix} 1 & r_{j,j+1} \\ r_{j,j+1} & 1 \end{pmatrix}, \tag{S.14}$$

with the standard Fresnel reflection and transmission coefficients

$$r_{j,j+1} = \frac{n_j - n_{j+1}}{n_j + n_{j+1}}, \qquad t_{j,j+1} = \frac{2n_j}{n_j + n_{j+1}}. \tag{S.15}$$

For the purpose of modelling spray-coated glucose, we take a Lorentz-Drude model for the refractive index around the resonance at $\nu = 2\pi \cdot 1.43\,\mathrm{GHz}$ (note that ω and ν are angular frequencies)

$$n_{gl}(\omega)^2 = \varepsilon_{gl}(\omega) = \varepsilon_{gl,\mathrm{back}} + \frac{\alpha_{gl}\nu^2}{\nu^2 - \omega^2 - i\gamma_{gl}\omega}, \tag{S.16}$$

with background permittivity $\varepsilon_{gl,\mathrm{back}} = 3.61$, oscillator strength $\alpha_{gl} = 0.028$, and linewidth $\gamma_{gl} = 2\pi \cdot 85\,\mathrm{GHz}$.

The total transmission matrix is then simply obtained by multiplying the matrices of the individual elements. In Fig. S2 we illustrate the potential advantages of hybrid cavity designs by comparing the transmission of a standard FP cavity to that of a hybrid architecture where the left mirror is replaced by an ideal, lossless MS. The hybrid architecture leads to a filtering out of all other resonances while simultaneously showing a reduction in linewidth around the resonance of interest (with a Fano-like profile and a zero of transmission located directly at the MS resonance).

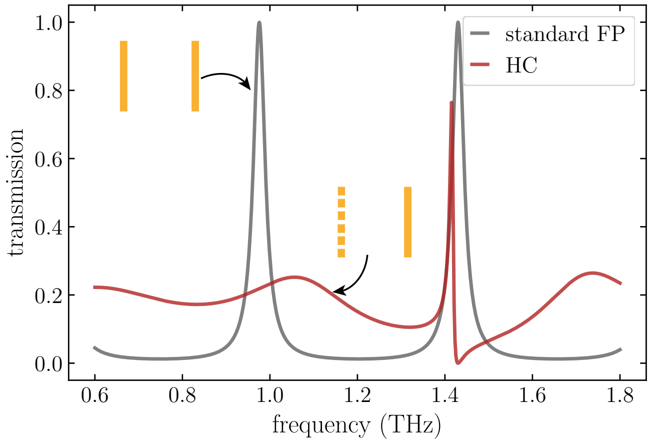

FIG. S2. Comparison between cavity transmission functions for a standard FP cavity with $\zeta = 2$ and a hybrid cavity design where one of the mirrors is replaced by a MS with linewidth $\gamma_m = 2\pi \cdot 50\,\mathrm{GHz}$ and resonance frequency $\omega_m = 2\pi \cdot 1.43\,\mathrm{THz}$. The cavity length for both architectures is $348\,\mu\mathrm{m}$.

S4. COUPLED-DIPOLES APPROACH FOR GLUCOSE-MS COUPLING

While the transfer matrix method describes the forward and backward propagation of plane waves through the structure, it does not per se include the near-field coupling of the glucose layer to the evanescent field of the MS. This coupling can be included by a coupled-dipoles model. To this end, one assumes that each vibrational dipole couples to the MS with a distance dependent rate $g_j \equiv g(\mathbf{r}_j)$. From the Tavis-Cummings Hamiltonian (Eq. (1) of the

manuscript), supplemented with the proper decay terms, one can then derive equations of motion for the amplitudes of the electric field of the MS and the jth molecular dipole in the glucose layer

$$\dot{\beta}_m = -(\gamma_m + \gamma_l + i\omega_m)\beta_m - i\sum_j g_j \beta_{gl}^{(j)} + \eta(t), \tag{S.17a}$$

$$\dot{\beta}_{gl}^{(j)} = -(\gamma_{gl} + i\nu)\beta_{gl}^{(j)} - ig_j\beta_m, \tag{S.17b}$$

where $\eta(t)$ describes some arbitrary input field probing the response of the MS and γ_{gl} is the linewidth of the glucose resonance. Solving this set of equations in Fourier domain, one can see that the coupling to the glucose gives rise to a modified MS response

$$\beta_m(\omega) = \frac{\eta(\omega)}{i(\omega_m - \omega) + \gamma_m + \gamma_l + \frac{\sum_j g_j^2}{\gamma_{gl} + i(\nu - \omega)}}, \tag{S.18}$$

and therefore also a renormalized polarizability of the MS

$$\zeta_{\text{hyb}}(\omega) = \frac{\gamma_m}{(\omega_m - \omega) - i(\gamma_m + \gamma_l) - i\frac{\sum_j g_j^2}{\gamma_{gl} + i(\nu - \omega)}}. \tag{S.19}$$

Assuming the field to fall off exponentially from the MS, we can write $g_j = g(z_j) = g_0 e^{-z_j/z_0}$ where z_0 is the penetration depth of the field into the glucose layer ($\approx 15\,\mu$m) and g_0 is the coupling strength directly at the MS (considering that we already averaged over the field distribution in the xy plane). Considering a total number of N molecular emitters coupling to the MS ($N = Ad_{gl}\rho$, with A the cross section of the glucose layer, d_{gl} the thickness of the glucose layer and ρ the density of molecules), the sum over the coupling strength can be turned into an integral

$$\sum_j g_j^2 = A\rho g_0^2 \int_0^{d_{gl}} dz\, e^{-2z/z_0} = g_0^2 \frac{N_{z_0}}{2}\left(1 - e^{-2d_{gl}/z_0}\right), \tag{S.20}$$

where N_{z_0} describes the number of emitters within the mode volume $N_{z_0} = Az_0\rho$. From this, we can define the effective coupling strength (Rabi frequency) as $g_{\text{eff}}(d_{gl}) = \sqrt{\sum_j g_j^2}$. In Fig. S3 we make use of Eq. (S.19) to fit the transfer matrix result to the experimentally measured transmission (parameters in caption).

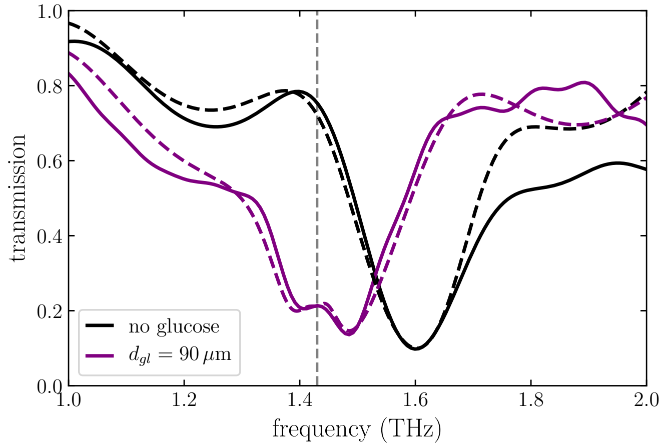

FIG. S3. Comparison of transmission between uncoated (black, $d_{gl} = 0\,\mu$m) and glucose-coated (purple, $d_{gl} = 90\,\mu$m) MS. The solid curves show the experimental results while the dashed curves show the transfer matrix fits which also take into account the effects of the Zeonor substrate. The MS is fitted with $\gamma_m = 2\pi \cdot 100\,$GHz, $\gamma_l = 2\pi \cdot 52\,$GHz, and we assume a coupling strength of $g_{\text{eff}} = 2\pi \cdot 45\,$GHz for $d_{gl} = 90\,\mu$m. The vertical dashed line shows the location of the bare glucose resonance.

S5. CONSTITUENT TRANSMISSION CURVES FOR H2

To visualize the evolution of the complex polaritonic response of the H2 cavity configuration, we plot as a waterfall plot in Fig. S4 the constituent transmission curves which were used to create the experimental transmission map of Fig.4. Here, one can observe how the polariton dips from the coupled metasurface become spaced further apart due to a Rabi splitting enhancement as a cavity mode is brought into overlap.

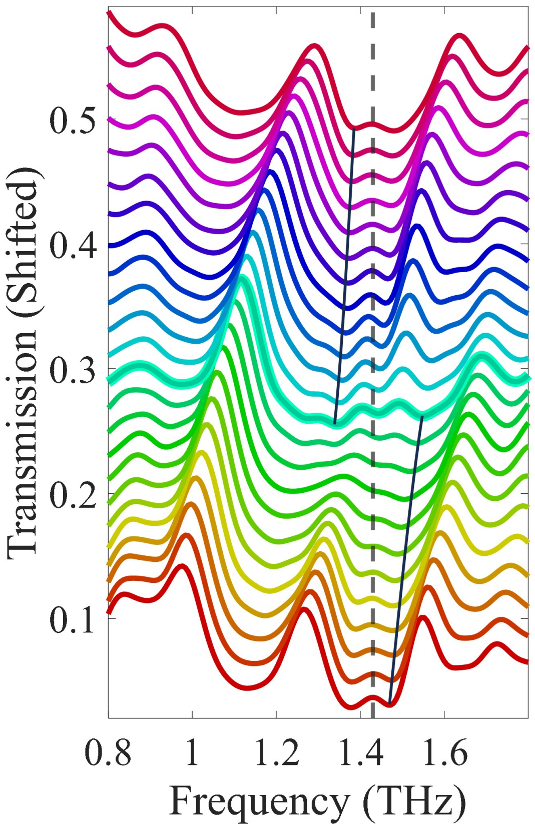

FIG. S4. Waterfall plot of the transmission curves that make up the experimental transmission map shown in Fig.4 for the H2 architecture. The relative cavity decreases from red (115 μm) to magenta (0 μm). The action of the overlapping photonic mode, to enhance the observed Rabi splitting, can be traced out in the spectral position of the polaritons (black curves). The grey dashed line indicates the glucose vibrational resonance frequency.

Away from the photonic resonance, considering for example the bottom curve, the transmission spectrum effectively shows a polaritonic splitting caused by the interaction of the glucose resonance at 1.43 THz (dashed line) with the MS plasmonic mode. This result agrees with the one shown in Fig. 3. As we decrease the cavity spacing and blueshift the photonic mode towards 1.43 THz, the upper polariton (UP) gradually shifts towards higher frequencies. A symmetrical behaviour with the lower polariton (LP) is observed after shifting the cavity mode to higher frequencies after 1.43 THz.

S6. EMPTY HYBRID CAVITY

To complement the experimental measurements on the H1 and H2 cavity architectures presented in the main text, we perform measurements of an empty hybrid cavity (no glucose) composed of a reflective metasurface facing a partially reflective metallic mirror. We discuss and plot the numerically calculated field distribution within such an empty hybrid cavity in Fig. 5(b) of the main text. In Fig. S2, we plot the transmission response of an empty hybrid cavity in comparison to the response of a Fabry-Perot cavity. Notably, the resonance of a hybrid cavity experiences a frequency shift and displays a Fano-like spectra. Fig. S5 shows three plots of experimental empty hybrid cavity measurements. In Fig. S5(a), we show the transmission of a standalone metasurface with a resonance frequency of 1.45 THz and the transmission of a planar mirror used in our hybrid cavity experiments. A vertical grey line is plotted

along the resonance frequency position of the metasurface. In Fig. S5(b), the hybrid cavity is formed and we show a transmission map of the experimental measurements. Around the overlap between the cavity mode and shifted metasurface resonance mode, we observe an anti-crossing feature. Prior works on empty hybrid cavity designs also demonstrate splitting just by the nature of having a plasmonic mode interacting with a cavity mode [S7]. In Fig. S5(c) we plot a few cavity spacings when the MS resonance is brought into overlap with the cavity mode resonance (green to red). We extend vertically the grey line showing the initial resonance frequency of the standalone MS.

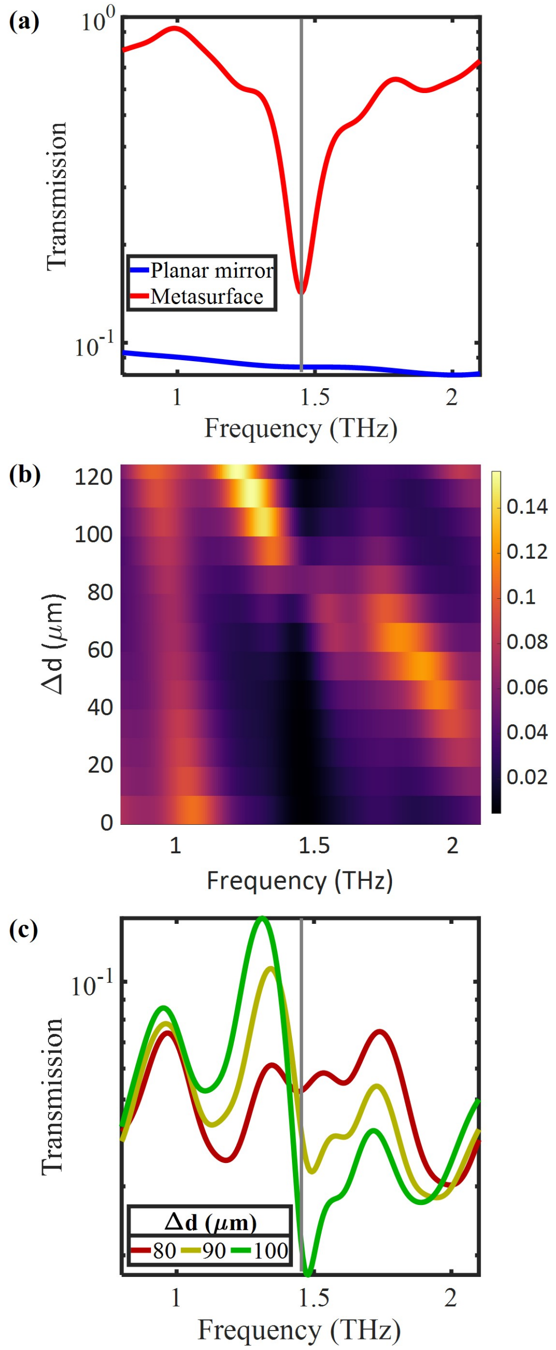

FIG. S5. Experimental measurements of the empty hybrid cavity. (a) comparison of the response of a planar mirror, created from sputtering gold on GaAs, and a notch resonator metasurface array with a resonance around that of glucose. (b) An empty hybrid cavity is constructed from the metasurface and planar mirror. A transmission map shows an anti-crossing feature around the overlap between a cavity mode and the metasurface resonance frequency. (c) A few measurements of varying relative cavity spacings are shown as a cavity mode is brought into spectral overlap with the metasurface mode.

S7. GLUCOSE FILLING FRACTION

As discussed in the main text, there are several parameters that determine the strength of a light-matter interaction. Namely, the coupling strength depends on the transition dipole moment, the orientation between the matter dipole and the photonic mode polarization, the zero-point electric field amplitude, the square root of the number of atoms/molecules, and the spatial distribution of the photonic mode. Furthermore, the zero-point electric field amplitude depends on the square root of the frequency and the inverse square root of the photonic mode volume. In our experiment, we target a vibrational mode of glucose to reach the regime of light-matter interactions. The transition dipole moment and the frequency of the molecular vibration are fixed parameters. Our polarization-insensitive MS/mirror design and orientation independent spray coating deposition technique allows us to neglect the dependence on orientation between the matter dipole and photonic mode polarization. Additionally, the photonic mode volume is fixed upon choosing a platform (MS, FP cavity, hybrid cavity). The only remaining controllable parameter is the number of glucose molecules within the mode volume of our photonic platform. Our spray coating technique presents an opportunity to fine-tune the density of glucose which can be coated within the mode volume of our various architectures. In this work, the MS and planar mirrors are coated with the same spray coating procedure and glucose batch, so the density should be relatively consistent across the experiments. Optical microscope images of the glucose coatings on the MS and planar mirror samples are shown in Fig. S6. The regions of the glucose coating are indicated with red dashed lines and the regions of the MS/planar mirror are indicated with blue dashed lines for clarity.

(a) (b)

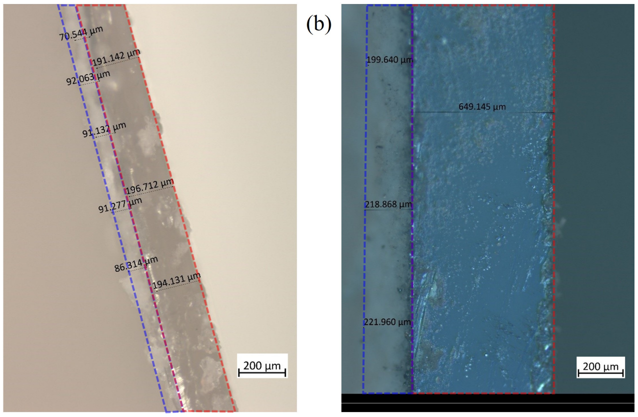

FIG. S6. Optical microscope images of the coating optical components. (a) Image of a cross resonator array MS on a Zeonor substrate ($\approx 188\,\mu$m, red dashed region) which is spray coated with a glucose coating (blue dashed region) that we estimate to be $\approx 90\,\mu$m. This is the MS that is utilized in the experiments of the paper. (b) Microscope image of a gold coated (9 nm) GaAs substrate ($\approx 650\,\mu$m, red dashed region) which is spray coated with glucose (blue dashed region) that we estimate to be $\approx 210\,\mu$m-thick.

Another method of comparison between the various architectures can be to monitor the filling fraction (FF) of glucose within the photonic mode volume. Here, as stated in the main text, we consider the FF to be

$$FF = \sqrt{\frac{\rho V}{V_{opt}}}, \tag{S.21}$$

where V_{opt} is the electromagnetic mode volume and ρ and V are the density and volume of glucose. Since our spray coating technique can be considered as uniform in area and thickness with respect to the scale of THz wavelengths, we express the FF to be a ratio of field integrals over the photonic mode distribution occupied by glucose to the total optical mode volume of the cavity.

$$FF \approx \frac{\int |E_{gl}|\,\mathrm{d}z}{\int |E_{opt}|\,\mathrm{d}z}, \tag{S.22}$$

where $|E_{gl}|$ represents the electromagnetic field magnitude within a 1D column that spans the region in the architecture filled with glucose and $|E_{opt}|$ is the total electromagnetic field inside the cavity which can potentially be coupled to glucose. We calculated the FF with numerical integration following FDTD simulations on the various

architectures which are plotted in Fig. S7. The glucose thickness and air spacing used for the simulations match what we measured/calculated experimentally. The glucose index is taken to be frequency independent with an index of $n_{gl} = 1.9$.

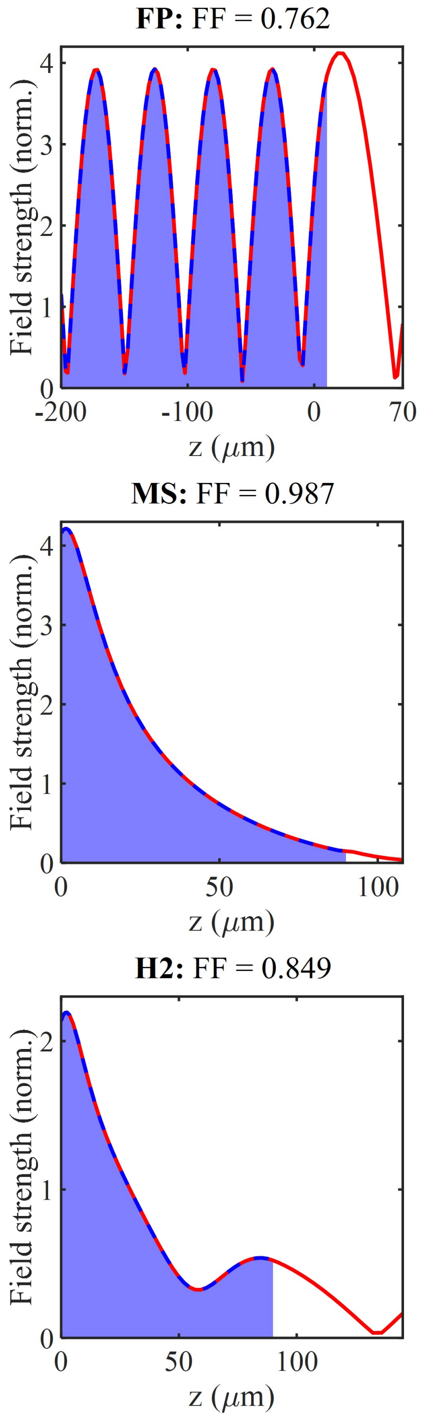

FIG. S7. Plots of 1D field magnitude simulations of the FP, MS, and H2 architectures containing glucose. The position of the 1D columns for the structures containing a MS is chosen at a position which is representative of the overall field contained within an xy plane above the MS. The total field across the electromagnetic mode volume is indicated by the red curve and the portion of the field within glucose is indicated by blue dashed lines and a filled blue area beneath the curve. The filling fraction for each architecture is calculated using (S.22).

The FP cavity and H1 architectures have the largest volumes of glucose (210 μm) but will have the smallest FF

because the electromagnetic field which interacts with glucose is not strongly confined to just the region of glucose. To reiterate, for H1, the MS effectively does not couple to glucose at all and just acts as a mirror which is why this architecture is equivalent to the FP cavity for the same glucose volume and density. The MS and H2 architectures have the strongest FFs with 90 μm of glucose. While the FF of H2 is lesser than the MS, the additional field enhancement within the glucose filled region of H2 allows for a substantially stronger light-matter interaction strength (200 GHz vs. 90 GHz) which we see in the experiment/calculation.

A. Overfilling a metasurface

For the MS in particular, we found it crucial not to overfill the resonator mode volume with glucose molecules to avoid a signal dominated by absorption. Below, we plot transmission spectra of a resonator as it is overfilled with glucose:

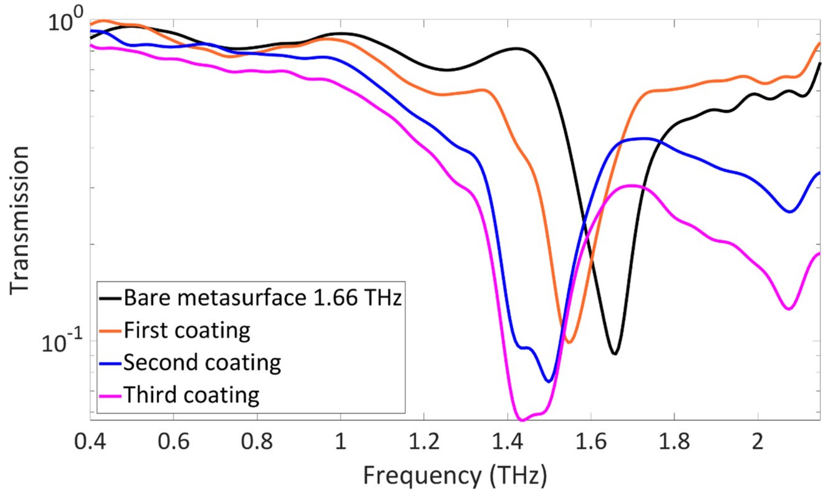

FIG. S8. Transmission spectra of a spray coated MS in which the mode volume is overfilled. A MS with a resonance frequency of 1.66 THz (black curve) is gradually coated with glucose and the resonance frequency begins to redshift (orange and blue curves). Once the plasmonic mode volume is completely filled with glucose we observe no more redshifting and the light-matter interaction cannot be further increased. Further coating the MS only causes the transmission to become absorption dominated (magenta curve).

In the paper, the MS we coat has an initial resonance frequency of 1.6 THz. We found that when we roughly fill the entire mode volume with glucose, the plasmonic resonance redshifts to about the vibrational resonance of glucose and couples strongly. In the plot above, we show a coated resonator with an initial resonance frequency of 1.66 THz. We show that continually coating this MS will eventually stop redshifting the plasmonic resonance and also not increase the light-matter coupling strength further. Coating the MS with too many glucose molecules results in linear absorption dominating the spectrum.

S8. GLUCOSE SPRAY COATING

Corresponding to the description in the main text, we show the extracted complex index of a glucose pellet at THz frequencies in Fig. S9(a). We also depict a schematic of the spray coating technique in Fig. S9(b).

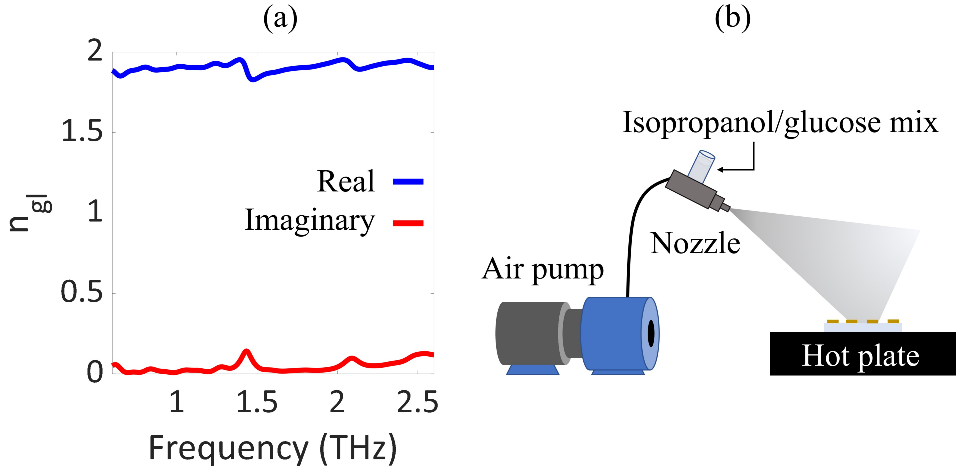

FIG. S9. (a) Extracted refractive index measurement of a glucose pellet with 300 μm thickness created using a hydraulic press. Glucose has an index of 1.9 (real part) at the targeted 1.43 THz vibrational mode. (b) Schematic of the spray coating technique. A suspension of sonicated glucose powder in isopropanol is air sprayed onto a sample which is placed onto a hotplate. The temperature of the hotplate is set to allow isopropanol to rapidly evaporate, leaving a layer of solid-state glucose on the sample.

[S1] F. J. García de Abajo, Rev. Mod. Phys. **79**, 1267 (2007).

[S2] L. Novotny and B. Hecht, *Principles of Nano-Optics* (Cambridge University Press, 2006).

[S3] T. R. Jensen, L. C. Kelly, A. A. Lazarides, and G. C. Schatz, Journal of Cluster Science **10**, 295 (1999).

[S4] M. S. Bin-Alam, O. Reshef, Y. Mamchur, M. Z. Alam, G. Carlow, J. Upham, B. T. Sullivan, J.-M. Ménard, M. J. Huttunen, R. W. Boyd, and K. Dolgaleva, Nature Communications **12**, 974 (2021).

[S5] M. Born, E. Wolf, A. B. Bhatia, P. C. Clemmow, D. Gabor, A. R. Stokes, A. M. Taylor, P. A. Wayman, and W. L. Wilcock, *Principles of Optics: Electromagnetic Theory of Propagation, Interference and Diffraction of Light*, 7th ed. (Cambridge University Press, 1999).

[S6] T. Mackay and A. Lakhtakia, Synthesis Lectures on Electromagnetics **1**, 1 (2020).

[S7] D. G. Baranov, B. Munkhbat, E. Zhukova, A. Bisht, A. Canales, B. Rousseaux, G. Johansson, T. J. Antosiewicz, and T. Shegai, Nature Communications **11**, 2715 (2020).
